# Polymer-Ceramic Composite Scaffolds: The Effect of Hydroxyapatite and β-tri-Calcium Phosphate

**DOI:** 10.3390/ma11010129

**Published:** 2018-01-14

**Authors:** Boyang Huang, Guilherme Caetano, Cian Vyas, Jonny James Blaker, Carl Diver, Paulo Bártolo

**Affiliations:** 1 School of Mechanical, Aerospace and Civil Engineering, University of Manchester, Manchester M13 9PL, UK; boyang.huang@postgrad.manchester.ac.uk (B.H.); cian.vyas@postgrad.manchester.ac.uk (C.V.); carl.diver@manchester.ac.uk (C.D.); 2 Graduate Program in Biomedical Sciences, Herminio Ometto University Center, Araras-SP 13607-339, Brazil; caetanogf@uniararas.br; 3 Bio-Active Materials Group, School of Materials, The University of Manchester, Manchester M13 9PL, UK; jonny.blaker@manchester.ac.uk

**Keywords:** 3D printing, hydroxyapatite, scaffold, tri-calcium phosphate

## Abstract

The design of bioactive scaffolds with improved mechanical and biological properties is an important topic of research. This paper investigates the use of polymer-ceramic composite scaffolds for bone tissue engineering. Different ceramic materials (hydroxyapatite (HA) and β-tri-calcium phosphate (TCP)) were mixed with poly-ε-caprolactone (PCL). Scaffolds with different material compositions were produced using an extrusion-based additive manufacturing system. The produced scaffolds were physically and chemically assessed, considering mechanical, wettability, scanning electron microscopy and thermal gravimetric tests. Cell viability, attachment and proliferation tests were performed using human adipose derived stem cells (hADSCs). Results show that scaffolds containing HA present better biological properties and TCP scaffolds present improved mechanical properties. It was also possible to observe that the addition of ceramic particles had no effect on the wettability of the scaffolds.

## 1. Introduction

Bone repair and regeneration is a common and complicated clinical problem. Bone, as a functionally smart tissue, is capable of healing and remodelling in the case of limited bone defects. However, bone is unable to heal itself when the defects are critical-sized [1,2]. Every year millions of people are affected with different types of bone diseases such as bone trauma, infections and tumours [3]. Current therapies for these bone defects are autografts, allografts, xenografts and artificial substitutes such as metals and bioceramics [4]. However, these treatments present several limitations. Autografts, which are harvested from one site and implanted into another site within the same individual, usually lead to a high risk of infection and hematoma [2]. Allografts, which are harvested from other donors, present a high risk of disease transmission, infection and rejection. Xenografts, which are harvested from one individual and transplanted into another individual of a different species, present reduced osteogenic properties, risk of immunogenicity, transmission of infection, zoonotic diseases, and poor clinical outcome. Finally, artificial metallic substitutes are associated with bone loss due to the stress shielding effect, while bioceramic substitutes are brittle and present low mechanical stability [5]. Moreover, the clinical efficacy of these grafts is limited in the case of large bone defects. Depending on the dimension and the biomechanical characteristics of the injured tissue, the implantation of synthetic grafts (scaffolds) produced using additive manufacturing technologies combined with biocompatible and biodegradable materials represents a viable approach for bone regeneration [6,7]. These grafts, usually porous structures with interconnected porosity, provide a temporary environment that guides the colonization, attachment and proliferation of either seeded or host cells, promoting tissue regeneration. They must also fulfil a series of requirements including biocompatibility, bioresorbability, appropriate stiffness, elasticity and surface properties [7]. Several 3D printing techniques such as extrusion-based, binder-jetting and powder-bed fusion technologies have been explored in order to produce biocompatible and degradable scaffolds for bone tissue engineering [6,8]. Among these techniques, extrusion-based processes, especially screw-assisted technologies, are particular relevant for bone regeneration due to their relatively low cost, low energy consumption, and the ability to process a wide range of materials from polymers to composites, guaranteeing, in the latter case, homogeneous mixtures. Several research groups used screw-assisted systems to produce poly-ε-caprolactone (PCL) scaffolds for bone regeneration [9,10,11]. Scaffolds with different topologies have been produced and assessed from both mechanical and biological points. However, PCL scaffolds present long degradation times, high hydrophobicity and poor bioactivity (osteointegration, osteoconduction and osteoinduction). To overcome these limitations and also to improve mechanical properties, PCL was combined with different inorganic materials such as hydroxyapatite (HA), tri-calcium phosphate (TCP), bioglass, and graphene [12,13,14,15,16,17,18,19].

Hydroxyapatite is widely used for different medical applications since it presents a similar mineral structure to natural bone [20,21]. It is an osteoconductive material that allows the formation of a high connection with the surrounding bone tissues [4,15,16,22,23]. However, major limitations are its brittleness and low mechanical stability, which prevents its use in large bone tissue regeneration. Moreover, its biodegradation and bioresorbability are quite low, as HA remains in the body for a long time after implantation. β-TCP is a form of TCP extensively used in tissue engineering [21]. It presents faster degradation than HA and good compatibility with cells. However, the individual usage of HA and TCP presents some limitations due to their brittleness, poor fatigue resistance and difficulty of shaping [22,23]. Several authors have investigated the use of 3D printed PCL/HA and PCL/TCP scaffolds [15,16,17,18,19], but so far there are no publications comparing the mechanical and biological performance of both. Therefore, this paper investigates the fabrication and performance of PCL scaffolds and PCL with different amounts of HA and TCP. Scaffolds were fabricated using a screw-assisted additive manufacturing system. The produced scaffolds were assessed for their morphological, physical, chemical, and biological properties.

## 2. Results and Discussion

### 2.1. Morphology

**Figure 1:**
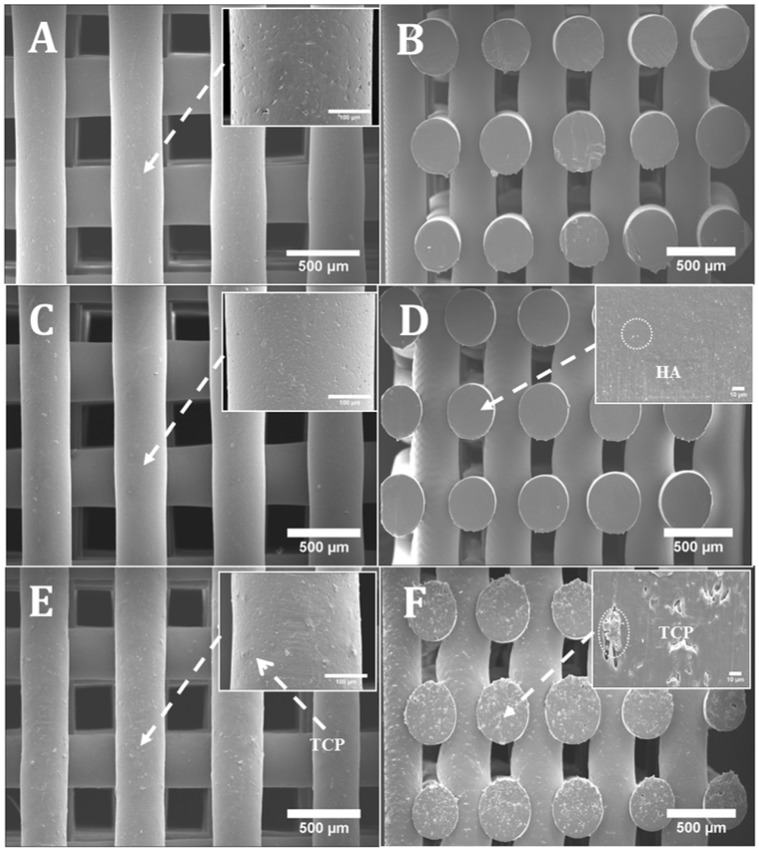
SEM (Scanning electron microscopy) images of (**A**) a poly-ε-caprolactone (PCL) scaffold top view and zoom-in view of filament; (**B**) a PCL scaffold cross section view; (**C**) a PCL/ hydroxyapatite (HA) 20 wt % scaffold top view and zoom-in view of filament; (**D**) a PCL/HA 20 wt % scaffold cross section view and zoom-in view of cut surface; (**E**) a PCL/ β-tri-calcium phosphate (TCP) 20 wt % scaffold top view and zoom-in view of filament; (**F**) a PCL/TCP 20 wt % scaffold cross section view and zoom-in view of cut surface.

Figure 1 shows SEM images (top and cross section views) of PCL, PCL/HA and PCL/TCP scaffolds. Results show that all scaffolds present a well-defined architecture with uniform pore distribution. The pore size of the PCL, PCL/HA (10% and 20%) and PCL/TCP (10% and 20%) scaffolds ranged between 287 and 317 µm, slightly smaller than the designed scaffold (350 µm), while the filament width ranged between 328 and 355 µm for a nozzle with an inner diameter of 330 µm (Table 1). These variations are due to the rheological characteristics of the melt, which depends on both material and processing conditions. The PCL and PCL/HA scaffolds (Figure 1A,C) seemed to have a smooth but micropitted surface, whereas the PCL/TCP scaffolds presented rough surfaces (Figure 1E). Micro-pores can be observed from the surface view of PCL filament (Figure 1A), while smaller but a larger number of pores were observed from the surface view of PCL/HA 20 wt % (Figure 1C). The formation of micro-pores might be due to the dispersion of ceramic aggregates on the fibre’s surface and also the collisions of the adjacent crystalline regions which were growing during solidification [24]. The addition of HA particles may reduce the size and increase the amount of PCL recrystallization zones, thus leading to smaller but more intense micro-porosity. Moreover, the addition of HA and TCP particles induces rheological changes on the melt, changing both the shear stress and shear rate on the interaction interface between the nozzle wall and flow materials, which may result in flow instabilities [25]. Therefore, the surface properties behaved differently between PCL scaffolds and composite scaffolds under the same process conditions. From Figure 1D,F, it can be observed that HA particles are nano-sized while TCP particles present micro-sized, which explains the rougher surface of the TCP scaffold compared to the HA scaffold.

### 2.2. Thermal Gravimetric Analysis (TGA)

**Figure 2:**
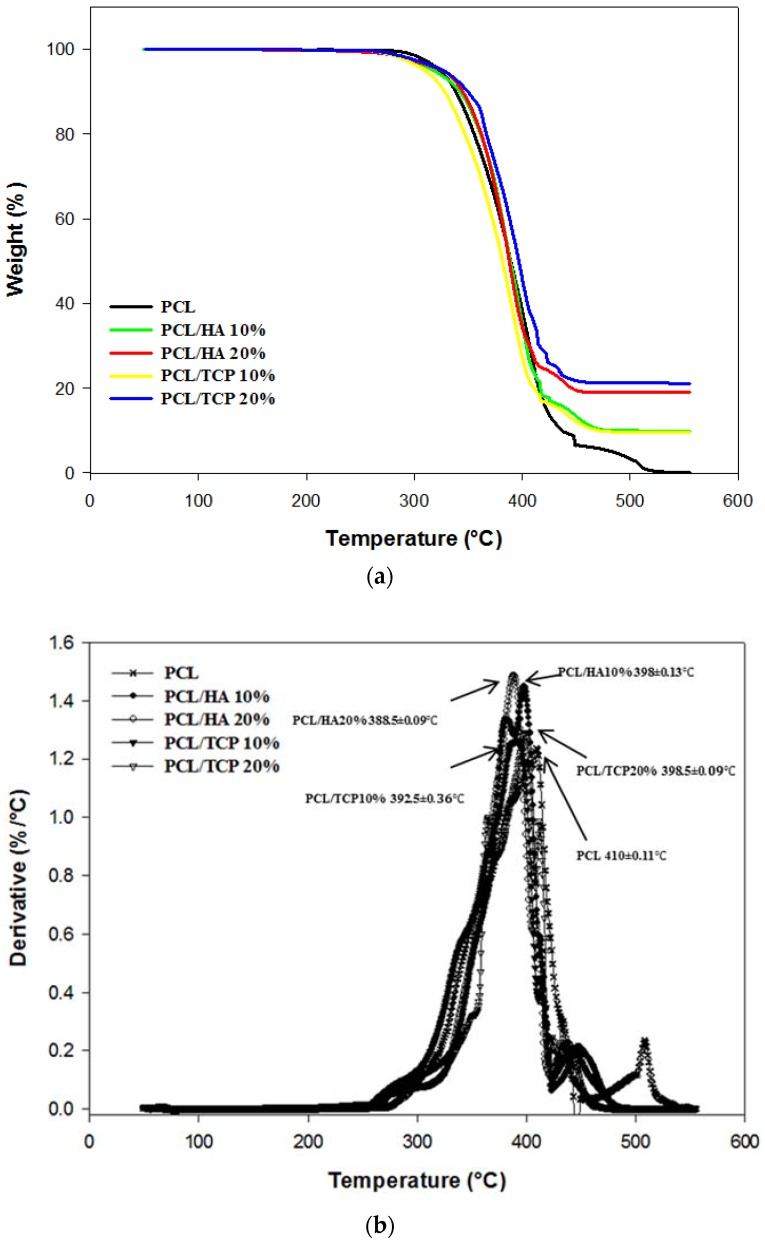
Thermal Gravimetric Analysis (TGA) curves (**a**) and derivative thermogravimetric (DTG) curves (**b**) for all scaffolds.

Thermal gravimetric analysis showed that the degradation temperature of the produced scaffolds ranged between 392.5 and 410 °C (Figure 2). Above this temperature, only the inorganic component (HA or TCP) remains. It was also observed that the addition of ceramic particles slightly reduced the degradation temperature. As the processing temperature used to produce the scaffolds was 90 °C, it is possible to conclude that the printing process did not induce material losses. Table 2 presents the residual amount of HA and TCP for samples prepared by melt blending. These results show that the melt blending process is an effective method to prepare composite blends for extrusion additive manufacturing. PCL pellets with HA concentrations of 9.95 and 18.95 wt %, and TCP concentrations of 9.64 and 21.13 wt % were produced.

### 2.3. Apparent Water-in-Air Contact Angle

**Figure 3:**
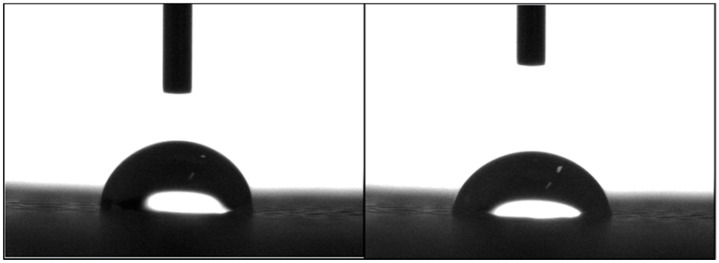
Water drop on a PCL scaffold filament at 0 s (**left**) and 20 s (**right**).

**Figure 4:**
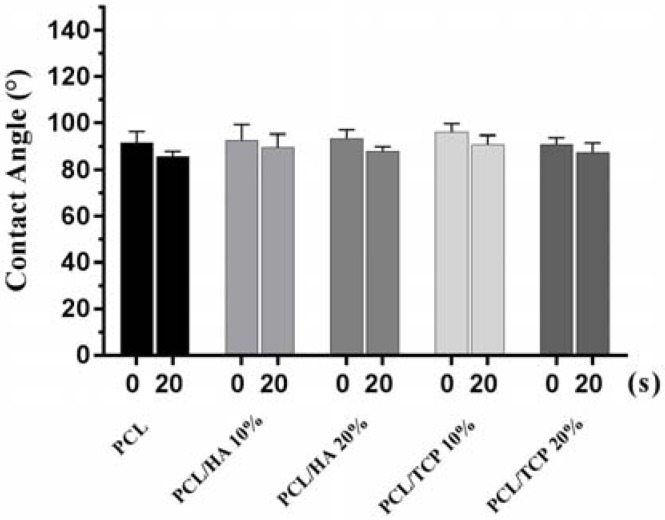
Contact angles obtained for all processed scaffolds at 0 s and 20 s, respectively.

Figure 3 shows two images illustrating the water droplet shape at different time points. Figure 4 shows the contact angles obtained for all processed scaffolds. It can be observed that the contact angle slightly decreased with time, indicating that water had been absorbed by the material. The results also show that the addition of HA or TCP had no effect on the hydrophobicity of the scaffolds.

### 2.4. Mechanical Analysis

**Figure 5:**
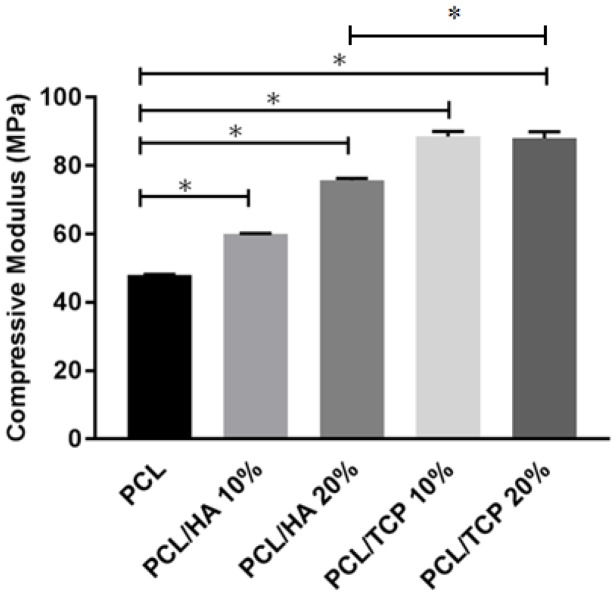
The compressive modulus of different materials. * Statistical evidence (p<0.05) using one-way analysis of variance (ANOVA) with Tukey test.

The compressive mechanical properties of PCL, PCL/HA (10% and 20%) and PCL/TCP (10% and 20%) scaffolds are presented in Figure 5. The results show an overall improvement in the compressive modulus due to the addition of ceramic particles. Additionally, for the same ceramic content, TCP seemed to have a more pronounced effect on the mechanical properties than HA. The addition of 20 wt % of ceramic particles enhanced the compressive modulus of PCL scaffolds from 48.08 ± 0.09 to 75.72 ± 0.57 MPa in the case of PCL/HA, or 88.07 ± 1.91 MPa in the case of PCL/TCP.

### 2.5. Cell Viability

**Figure 6:**
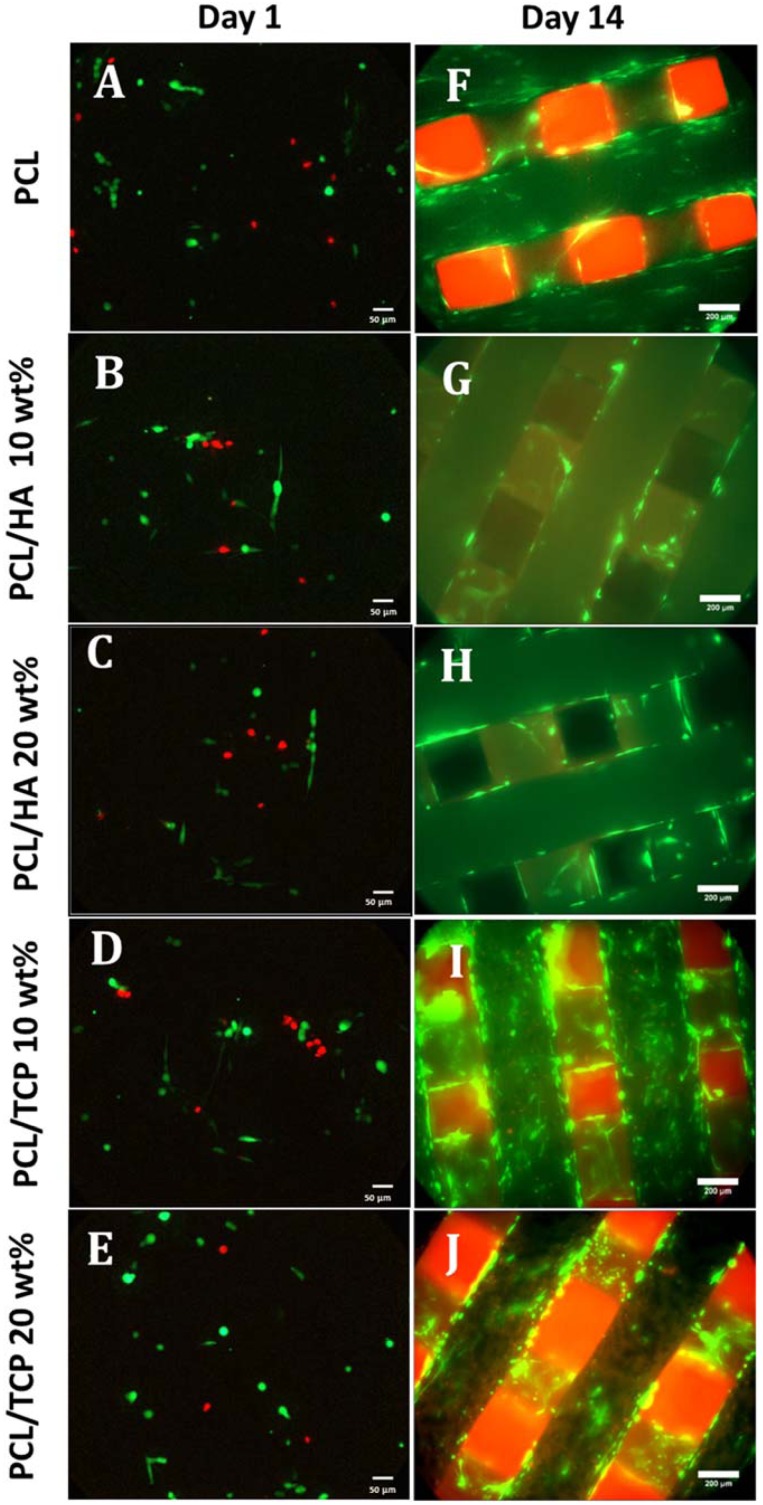
Live (green) and dead (red) cells on PCL, PCL/HA 10 wt %, PCL/HA 20 wt %, PCL/TCP 10 wt %, and PCL/TCP 20 wt % at day 1 (**A**–**E**, respectively, 20× magnification) and day 14 (**F**–**J**, respectively, 10× magnification).

**Figure 7:**
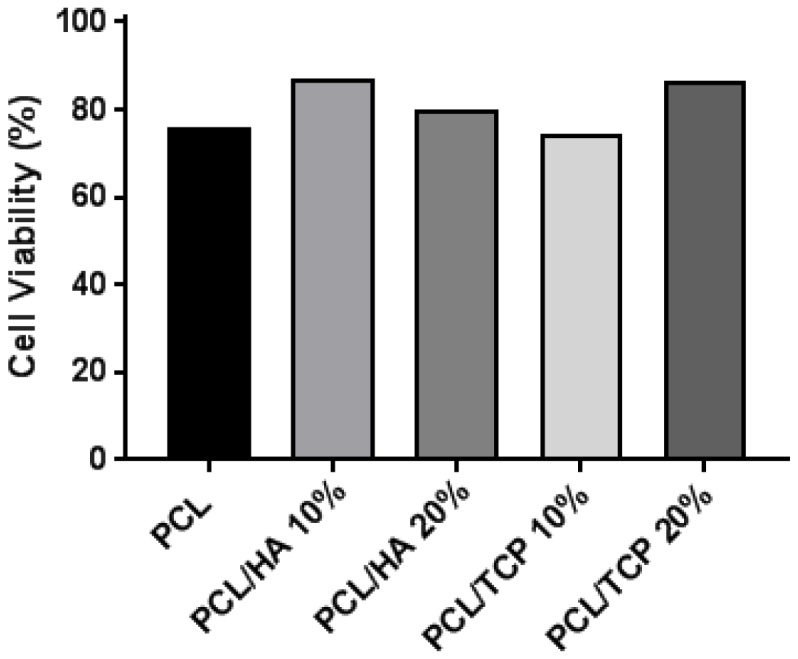
Cell viability (%) for all scaffolds at day 1.

Figure 6 shows the live (green) and dead (red) cells after seeding the scaffolds at day 1 (Figure 6A–E) and day 14 (Figure 6F–J), and Figure 7 shows the cell viability for all scaffolds at day 1. Results show that all samples presented good biocompatibility since cell viability was approximately 80%. According to Figure 6A–E, after 24 h of cell seeding, it was possible to observe live cells with spindle shape morphology attached to the scaffold filament. After 14 days (Figure 6F–J), the cells were filling the surface of the fibres and spreading between the fibres and layers. Some dead cells can be observed, however, the majority of cells were alive.

### 2.6. Cell Attachment and Proliferation

**Figure 8:**
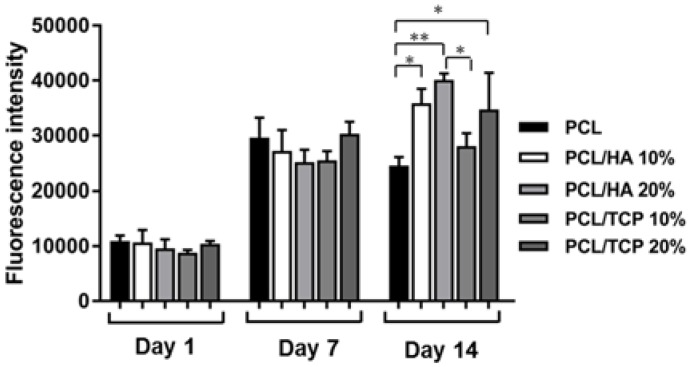
Fluorescence intensity for all different scaffolds at day 1, day 7, and day 14. p*<0.05 and p**<0.01 using one-way analysis of variance (ANOVA) with Tukey test.

**Figure 9:**
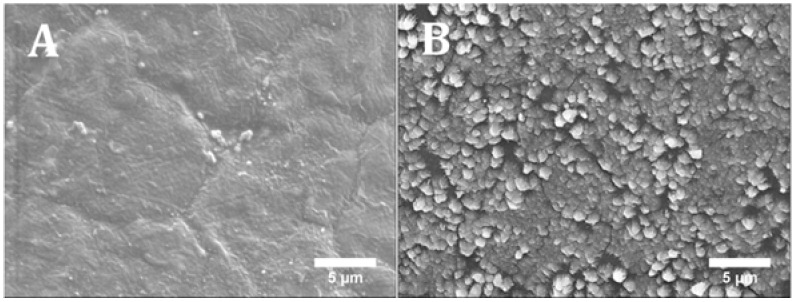
SEM images of PCL/HA 20 wt % surface (**A**) without cells (printed scaffold) and (**B**) with cells after 14 days (cell cultured scaffold).

Figure 8 shows the results of cell proliferation of human adipose derived stem cells (hADSCs)at day 1, day 7, and day 14, assessed using the Alamar Blue Assay. According to the results, the fluorescence intensities for all scaffolds presented no significant differences at day 1, while a sharp increase in fluorescence intensity was observed for all samples at day 7, indicating a large quantity of cell metabolic activity, which implies an increase in the cell number due to cell proliferation. Results also show that the addition of HA particles and TCP particles did not influence the biological behaviour compared to PCL at early stage of cell culture. However, at day 14, higher fluorescence intensities were observed for PCL/HA 20 wt % and PCL/TCP 20 wt % compared to PCL. Results also show that a higher content (wt %) of HA and TCP led to an increase in cell proliferation. Moreover, an apatite layer was formed on the PCL/HA 20 wt % scaffold surface (Figure 9), which correlates with Bohner et al.’s findings [26]. The HA surface provides nucleating sites for the precipitation of apatite crystals, which potentially have favourable biological effects by providing trace ions. No significant differences were observed on the surface of TCP scaffolds before and after cell culture. This may be attributed to the different mechanisms of nucleation and growth of crystals on HA and TCP surfaces (HA provides apatite nuclei for the formation of apatite crystals) [27]. In summary, results seem to indicate that increasing the inorganic content increases the bioactivity characteristics of the scaffolds and that HA allows an increased rate of cell proliferation compared to TCP.

### 2.7. Cell Morphology

**Figure 10:**
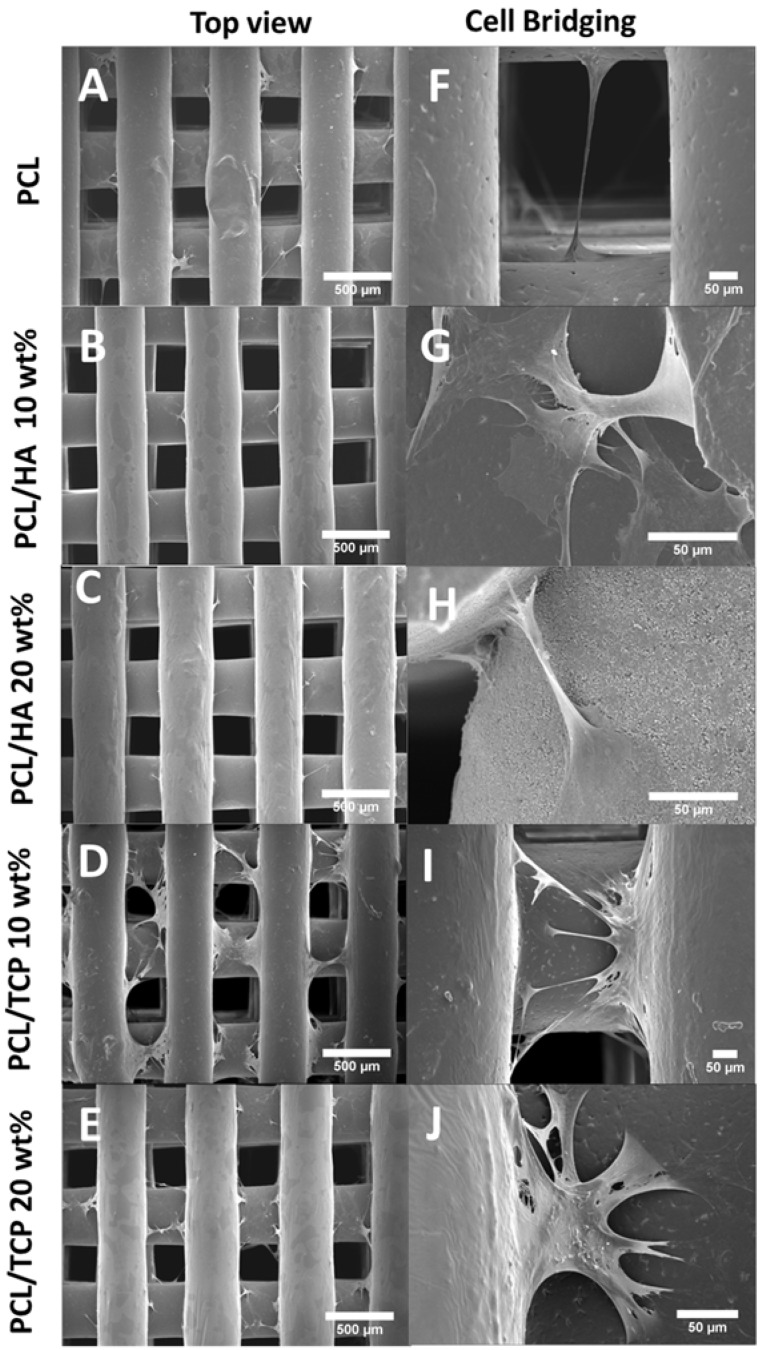
SEM images of cell attachment (**A**–**E**) and cell bridging (**F**–**J**) on the PCL, PCL/HA 10 wt %, PCL/HA 20 wt %, PCL/TCP 10 wt %, and PCL/TCP 20 wt % scaffolds, respectively.

**Figure 11:**
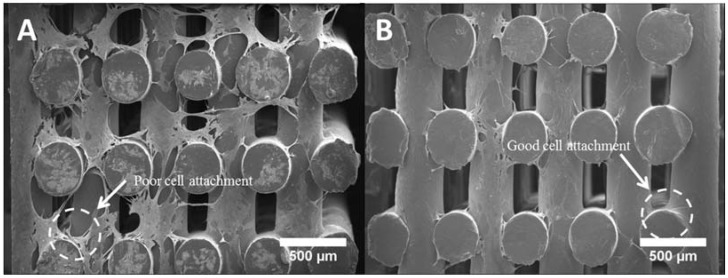
Cross section of (**A**) a PCL scaffold and (**B**) a PCL/HA scaffold after 14 days of cell culture.

Figure 10 shows the cell morphology of all scaffolds at day 14. It can be seen that all scaffolds provided a suitable environment for cell adhesion, migration and proliferation, since extensive cells were observed throughout the scaffolds. According to Figure 10A–E, the cells expanded along the fibre surface and started to bridge between layers and fibres (Figure 10F–J). A large uncovered area on the PCL scaffolds surface can also be observed (Figure 10A,F), indicating the formation of a thinner layer of cell sheets compared to the bioceramics scaffolds. PCL/HA 20 wt % seemed to present thicker cell sheets than PCL/HA 10 wt %, which agrees with the results of the Alamar Blue assay. PCL/TCP scaffolds presented a similar trend with increasing concentrations of ceramic as TCP particles were obscured on the surface of the PCL/TCP 20 wt % fibre, indicating that cells were covering the surface. Moreover, PCL/HA scaffolds presented less cell bridging phenomena, especially at cross points between layers (Figure 11). This may imply stronger cell adhesion on the PCL/HA scaffolds at a later cell culture period.

## 3. Materials and Methods 

### 3.1. Materials

Poly-ε-caprolactone (PCL) (CAPA 6500, Mw = 50,000 Da) in the form of 3 mm pellets was obtained from PerstorpCaprolactones (Cheshire, UK). Hydroxyapatite (HA) (Mw = 502.31 g/mol, Mp = 1100 °C) in the form of nanopowder (<200 nm particle size) and tricalcium phosphate (TCP) (Mw = 310.18 g/mol, Puriss ≥ 98%) in the form of sintered powder were both supplied by Sigma-Aldrich (St. Louis, MI, USA). PCL/HA and PCL/TCP composite materials with different concentrations were prepared using melt blending. The PCL pellets were heated up to 90 °C in a grounding bowl for 20 min and then manually mixed for at least 30 min. During the mixing process, HA and TCP particles were added slowly into the melted PCL matrix to produce different blends.

### 3.2. Scaffold Fabrication

**Figure 12:**
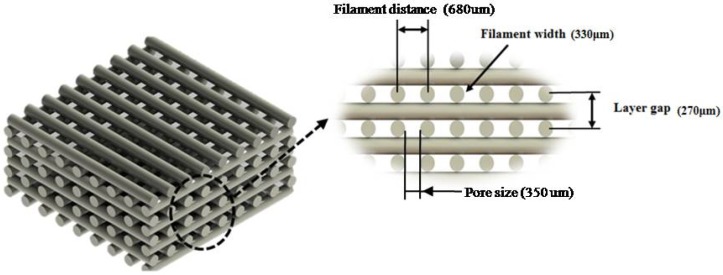
0/90° lay-down pattern and design characteristics.

PCL, PCL/HA (10 and 20 wt % of HA) and PCL/TCP (10 and 20 wt % of TCP) scaffolds were fabricated using the 3D Discovery (regenHU, Villaz-Saint-Pierre, Switzerland), which is a screw-assisted additive manufacturing system. Scaffolds were fabricated by applying a 0/90° lay-down pattern (see Figure 12) and using a melting temperature of 90 °C, a feed rate of 20 mm/s and a screw rotational velocity of 22 rpm.

### 3.3. Scaffold Morphology

Scanning electron microscopy (SEM), Hitachi S-3000N (Hitachi, Tokyo, Japan), was used to assess the morphology of the produced scaffolds. Relevant morphological characteristics were obtained using the software ImageJ and at least fifteen measurements were considered in order to assess each design characteristic.

### 3.4. Thermal Gravimetric Analysis (TGA)

The onset of thermal degradation and ceramic content in the scaffolds was assessed using a TA Instruments Q500 TGA (TA Instrument, New Castle, DE, USA) equipped with an evolved gas analysis furnace. TGA was performed on PCL scaffolds as controls, and PCL/HA and PCL/TCP composite scaffolds. Scans were performed in an air atmosphere (flow at 60 mL/min) with a temperature ranging from room temperature to 560 °C at a rate of change of 10 °C/min. Measurements were taken using a sample mass ranging from 11 to 17 mg in platinum pans, performed in triplicate.

### 3.5. Apparent Water-in-Air Contact Angle

The water contact angle is a significant parameter to indicate the wettability of the material surface and the interaction between the scaffolds and cells. The contact angle enables the understanding of the hydrophilic/hydrophobic characteristics of the structure. A contact angle below 90° means a hydrophilic surface while a contact angle value above 90° corresponds to hydrophobic surfaces.

Static contact angle measurements were performed using the equipment OCA 15 (Data Physics, San Jose, CA, USA) and a droplet of deionised water (4 μL of volume drop, 1 μL/s of velocity) was ejected onto the top surface of a scaffold filament. For each condition, at least four measurements were performed using the sessile drop method. The drop shape was recorded with a high speed framing camera. Measurements were performed at the time the droplet was dropped onto the scaffold’s fibre and after a static time of 20 s.

### 3.6. Mechanical Analysis

Compression tests were performed on the 3D printed scaffolds in order to evaluate the effect of the composite materials and ceramic contents on the mechanical properties. After fabrication, each scaffold was cut into smaller samples of appropriate dimensions (i.e., length of 4.0 mm, width of 4.0 mm and height of 5.0 mm). All tests were carried out on scaffolds in a dry state at a rate of 1 mm/min up to a strain value of 0.4 mm/mm, using an INSTRON 4507 testing system (High Wycombe, UK) equipped with a 1 kN load cell and performed three times.

### 3.7. Cell Proliferation

Human adipose derived stem cells (hADSCs) (STEMPRO^®^, Invitrogen, Carlsbad, CA, USA) was used to investigate the cytocompatibility of the scaffolds. MesenPRO RS^TM^ Basal media, 2% (*v*/*v*) growth supplement, 1% (*v*/*v*) glutamine, and 1% (*v*/*v*) penicillin/streptomycin (STEMPRO^®^, Invitrogen, Carlsbad, CA, USA) were used for the purpose of maintaining hADSCs. Phosphate buffered saline (PBS) adjusted to a PH of 7.4 was obtained from Sigma-Aldrich (Gillingham, UK). Cell proliferation was tested using the Resazurin assay, also known as the Alamar Blue assay, which produces fluorescent signals detected by a microplate reader (Infinite 200, Tecan, Männedorf, Switzerland). Resazurin (7-hydroxy-10-oxido-phenoxazin-10-ium-3-one) dye was used to measure cytotoxicity and proliferation. Cells are able to reduce resazurin to resorufin intracellularly by mitochondrial enzyme activity based on their cellular metabolic activity. AlamarBlue assay was used at day 1, 7, and 14. All scaffolds were sterilized using 80% ethanol for 2 h, rinsed by PBS three times, and air-dried overnight in a sterile laminar flow cabinet. The scaffolds were transferred into 24 well plates and kept in the incubator prior to the cell seeding. 500 µL of cell suspension containing 5 $\times 10^{4}$ cells was added into each well at day 0. A tissue culture plastic control with the same number of cells was used in order to evaluate the cell seeding efficiency after day 1. For day 1, all scaffolds were transferred to new plates and 500 µL of fresh cell culture media was added before 50 μL of Alamar Blue was added into each well and the well-plates were placed for 4 h in an incubator. After 4 h, 150 µL of media was extracted from each well and delivered into a 96 well plate and read using a microplate reader. The scaffolds were washed by PBS twice to remove Alamar Blue for the sake of the next measurement. New cell culture media was added to each well and well-plates were placed in the incubator. Cell culture media was changed every 3 days. A similar procedure was used for the other days.

### 3.8. Cell Morphology

Cell Morphology was investigated by using SEM (Hitachi S-3000N, Hitachi, Tokyo, Japan). The seeded scaffolds at day 14 were rinsed twice in PBS, fixed in formalin solution (10%) for 30 min and then dehydrated in a series of ethanol concentrations (50%, 60%, 70%, 80%, 90% and 100%), sequentially for 15 min each, with the 100% concentration repeated. This was followed by dehydration using hexamethyldisilazane (HMDS, Sigma-Aldrich, Gillingham, UK) and ethanol in a 50:50 mixture for 15 min, before using 100% HMDS for 15 min. This was allowed to dry for 24 h before coating with platinum and observation with SEM.

### 3.9. Live/Dead Assay

Cell viability was assessed using a Live/Dead Assay Kit (Thermo Fisher Scientific, Waltham, MA, USA) at day 1 and day 14. Two reagents, Calcein-AM and Ethidium homodimer (EthD-1), were prepared by following the manufacturer’s instructions. 2 µM of Calcein-AM and 4 µM of EthD-1 were prepared in PBS respectively. All samples were rinsed in PBS twice prior to adding 500 μL of Calcein-AM and EthD-1 solutions. The samples were then incubated for 25 min in a cell culture incubator. An inverted fluorescence microscope (Leica DMI6000 B, Leica Microsystems, Wetzlar, Germany) was used to investigate the live and dead cells. The images were taken alongside Z stacks, and analyzed using ImageJ software to produce the results of day 1. However, it was not applicable for calculating cell viability at day 14 since the cell density was too confluent.

### 3.10. Statistical Analysis

The statistical analysis was performed using one-way analysis of variance (ANOVA) with Tukey test. Differences were considered statistically significant at p*<0.05 and p**<0.01. GraphPad Prism software (Graphpad Software Inc., San Diego, CA, USA) was used in this research.

## 4. Conclusions 

3D porous scaffolds of PCL, PCL/HA and PCL/TCP were fabricated using a screw-assisted extrusion additive manufacturing process. The pore size of PCL, PCL/HA, PCL/TCP scaffolds ranged between 287 and 317 µm. The PCL and PCL/HA scaffolds had smoother surfaces than those of the PCL/TCP scaffolds. TGA analysis shows that the melt blending method used to prepare the PCL/HA and PCL/TCP pellets is a simple and effective method, as the exact concentration of ceramic material in the scaffolds was similar to the designed parameters. The results also show that the optimal processing temperature (90 °C) does not induce any degradation of the polymer (no weight loss was observed). The addition of ceramic material had no influence on the hydrophilic characteristics of the PCL scaffolds. The addition of HA and TCP particles enhanced the mechanical properties, with the compressive modulus of TCP scaffolds being higher than those of HA. In addition, cell proliferation of hADSCs shows that the PCL/HA (20%) and PCL/TCP (20%) scaffolds presented an increased proliferation rate than the PCL scaffolds at day 14 although no differences were observed for all samples at day 1 and 7. Results suggest that the addition of HA, compared to TCP, improves the biological performance of produced scaffolds but with lower mechanical properties. The results suggest that an ideal composite scaffold for bone regeneration should be composed of a mixture of HA and TCP.

## Figures and Tables

**Table 1:** Morphological parameters for PCL, PCL/HA and PCL/TCP scaffolds.

	Pore Size (µm)	Filament Width (µm)	Layer Gap (µm)
PCL	287.76 ± 17.68	355.01 ± 18.16	258.99 ± 19.26
10% PCL/HA	314.54 ± 4.80	328.91 ± 4.90	155.10 ± 11.90
20% PCL/HA	305.80 ± 8.30	346.60 ± 7.00	181.70 ± 11.90
10% PCL/TCP	305.80 ± 12.80	349.50 ± 14.50	171.00 ± 9.70
20% PCL/TCP	317.47 ± 5.27	334.83 ± 15.43	176.10 ± 12.32

**Table 2:** Concentration of HA and TCP on the composite scaffolds.

	Designed Concentration (wt %)	Measured Concentration (wt %)	Degradation Temperature (°C)
PCL	0	0.00	410.00 ± 0.11
PCL/HA	10	9.95 ± 0.13	398.00 ± 0.13
PCL/HA	20	18.97 ± 0.16	388.50 ± 0.09
PCL/TCP	10	9.64 ± 0.26	392.50 ± 0.36
PCL/TCP	20	21.13 ± 0.35	398.50 ± 0.09
